# Analysis of Grindability and Surface Integrity in Creep-Feed Grinding of High-Strength Steels

**DOI:** 10.3390/ma17081784

**Published:** 2024-04-12

**Authors:** Youkang Yin, Ming Chen

**Affiliations:** State Key Laboratory of Mechanical System and Vibration, School of Mechanical Engineering, Shanghai Jiao Tong University, Shanghai 200240, China; yk-yin120526@sjtu.edu.cn

**Keywords:** high-strength steel, creep-feed grinding, surface integrity, grindability

## Abstract

Creep-feed grinding of high-strength steel is prone to excessive wheel wear and thermal damage defects, which seriously affects the service performance of parts. To solve the above-mentioned issue, a creep-feed grinding test was carried out on high-strength steel using SG and CBN abrasive wheels. The grindability of high-strength steel was scrutinized in terms of grinding force, machining temperature and grinding specific energy. Moreover, the effects of operation parameters and grinder performances on the surface integrity of the workpiece such as surface morphology, roughness, residual stress and hardness were rigorously studied. The results indicate that, when the instantaneous high temperature in the grinding area reaches above the phase transition temperature of the steel, the local organization of the surface layer changes, leading to thermal damage defects in the components. The outstanding hardness and thermal conductivity of CBN abrasives are more productive in suppressing grinding burns than the high self-sharpening properties of SG grits and a more favorable machining response is achieved. The effects of thermal damage on the surface integrity of high-strength steel grinding are mainly in the form of oxidative discoloration, coating texture, hardness reduction and residual tensile stresses. Within the parameter range of this experiment, CBN grinding wheel reduces grinding specific energy by about 33% compared to SG grinding wheel and can control surface roughness below 0.8 µm. The weight of oxygen element in the burn-out workpiece accounts for 21%, and the thickness of the metamorphic layer is about 40 µm. The essential means of achieving burn-free grinding of high-strength steels is to reduce heat generation and enhance heat evacuation. The results obtained can provide technical guidance for high-quality processing of high-strength steel and precision manufacturing of high-end components.

## 1. Introduction

High-strength steel is widely used in aerospace, automotive, marine and energy equipment due to its excellent wear resistance, corrosion resistance and high temperature resistance [1,2]. Higher strength and toughness can be achieved through reasonable chemical composition ratio and heat treatment process [3,4]. Among them, 30Cr2Ni4MoV steel is adopted as an important raw material for precision parts or other drive systems due to its favourable properties. The final critical step of the surface creep-grinding process is usually performed on the precision workpieces that have already been formed in order to control the surface integrity and to achieve the desired service performance of the product [5,6].

Unfortunately, in the current precision machining industry, there is a wide range of high-hardness materials or other special materials difficult to machine. In practice, poor grinding processes can lead to significant substandard quality of prototypes or severe grinding surface burns, resulting in extremely high reject rates [6,7]. To solve the above engineering problems, scholars have never stopped researching the suppression of grinding thermal damages in difficult-to-machine metal materials such as high-strength steels for many years. Fruitful scientific research results have also been achieved.

Typically, Sinha et al. [8] conducted a comparative analysis of the inhibition properties of grinding burns on SiC and Al_2_O_3_ grinding wheels from the perspective of surface integrity and surface behaviour of the machined parts. The high temperature dissociation of SiC abrasives and the chemical reaction with metallic materials were found to be the dominant factors responsible for the severity of damages. Lin et al. [9] established a relationship between the state of the steel grinding surface, grinding burns and grinding temperature. The authors concluded that the essence of grinding burns on metal specimens is that the grinding temperature exceeds the austenitization temperature of the material, resulting in increased oxidation of the surface of the workpiece and the accumulation of nonferrous oxides, which leads to the appearance of a white etching layer. Similarly, Österle et al. [10] found a surface white layer structure with embedded equiaxial crystals when grinding metallic materials. The authors attributed this structure to the localized high temperatures in the arc zone exceeding the melting point of the material and the sharp cooling of the grinding fluid. Although the hardness of the surface white layer is higher than that of the workpiece substrate, it is brittle and prone to cracking, so this structure should be avoided at all costs. Douglas et al. [11] reported that the MQL techniques not only can match the lubrication effect of the pouring method but also eliminate the environmental pollution due to the biodegradable effect. Unfortunately, the cooling performance of this technology is poor and the high processing temperatures lead to oil film rupture, desorption and even oxidative failure.

It can be seen that the current research hotspots are mostly focused on how to optimize the processing conditions to inhibit grinding thermal damage. However, there are few reports on the formation mechanisms of thermal damage in creep-feed grinding of high-strength steel and the impact on surface integrity, which makes it impossible for the industry to put forward effective detection indexes and inhibition means of thermal damages in grinding. In addition, the SiC abrasive wheels commonly used in high-strength steel grinding are prone to adhesion and clogging [12,13]. In recent years, the introduction of microcrystalline corundum abrasives and cubic boron nitride grinding wheels that are cost-effective has become an important development direction for the application of high-strength steels and other hard-to-cut materials for large-load grinding [4].

The aim of this paper is to fill the above technology gap based on the current state of research. Therefore, this project carries out the creep-feed grinding test of 30Cr2Ni4MoV steel and reveals the effect mechanisms of working conditions on the grindability of high-strength steel by studying the role of process parameters on the procedure parameters, such as grinding force, grinding temperature, specific energy, etc. In addition, the influence of grinding thermal damage of high-strength steel on the surface morphology, residual stress, microhardness, surface energy spectrum and organizational structure of the specimen is discussed. The main performances in the properties of high-strength steels when grinding defects occur are identified. The innovation of this article is to propose effective detection and suppression methods based on the formation mechanism of thermal damage in high-strength steel grinding. The research results have profound guiding significance for the promotion of precision grinding technology for hard-to-machine materials such as high-strength steel.

## 2. Experimental Setup

### 2.1. Grinding Equipment, Grinding Tools and Workpieces

**Figure 1:**
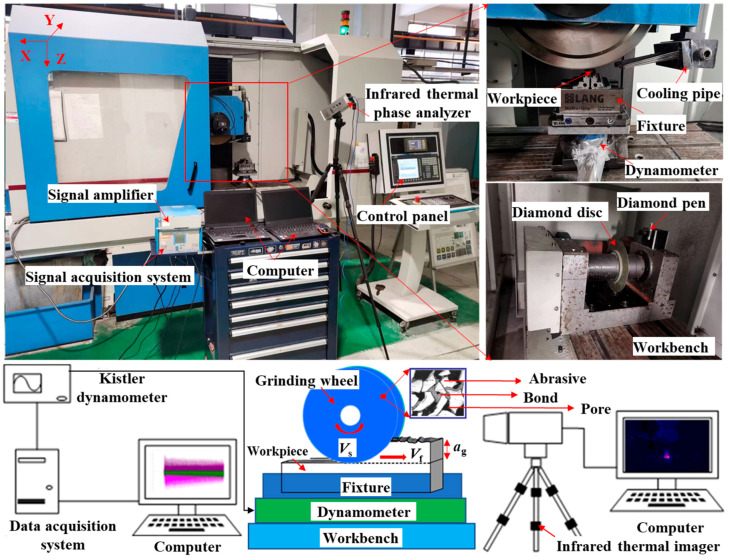
Layout of grinding experiment site and schematic diagram of machining principle.

As detailed in Figure 1, the grinding test was conducted on MKL7132 CNC power grinder. A Siemens 802D operating system was adopted, and the diamond pen/disk were carried out by Y and Z axis linkage for dressing. The test pieces of the 30Cr2Ni4MoV high-strength steel were stably fastened by a LANG fixture, and a dynamic force measuring system including a KISTLER 9272 piezoelectric dynamometer, a 5070 A charge amplifier and a data acquisition system was employed to record the force signals during the grinding process where the sampling frequency was set to 2000 Hz.

30Cr2Ni4MoV is a common engineering structural steel, and its composition and thermo-mechanical properties are listed in Table 1 and Table 2, respectively. The length × width × height of the specimen is 15 mm × 10 mm × 10 mm. It is worth emphasizing that 30Cr2Ni4MoV is an ultra-pure steel for forgings. The material is characterized by low fatigue sensitivity and crack extension rate, high fracture toughness, etc., which makes an important contribution to guaranteeing the safety and stability of the turbine. Given the excellent mechanical properties and wide application range of 30Cr2Ni4MoV steel, choosing this material as the workpiece to be ground has strong representative significance.

The grinding wheels were fabricated from Al_2_O_3_ abrasive and CBN abrasive, respectively, and their thermal-mechanical performance parameters are shown in Table 2. The characteristic parameters of the grinding tool are detailed in Table 3.

### 2.2. Grinding Process Parameters

As illustrated in Table 4, the grinding trial adopts a creep-feed machining mode and analyzes the effects of workpiece feed rate (*V*_f_), grinding depth (*a*_p_), wheel speed (*V*_s_) and abrasive properties on the operating procedure parameters and surface integrity by controlling a single variable. Among them, procedure parameters refer to grinding force (*F*), machining temperature (*T*) and specific energy (*E*_s_) used to discuss the effect of process parameters on the grindability of the specimen.

### 2.3. Detection of Surface Integrity

**Figure 2:**
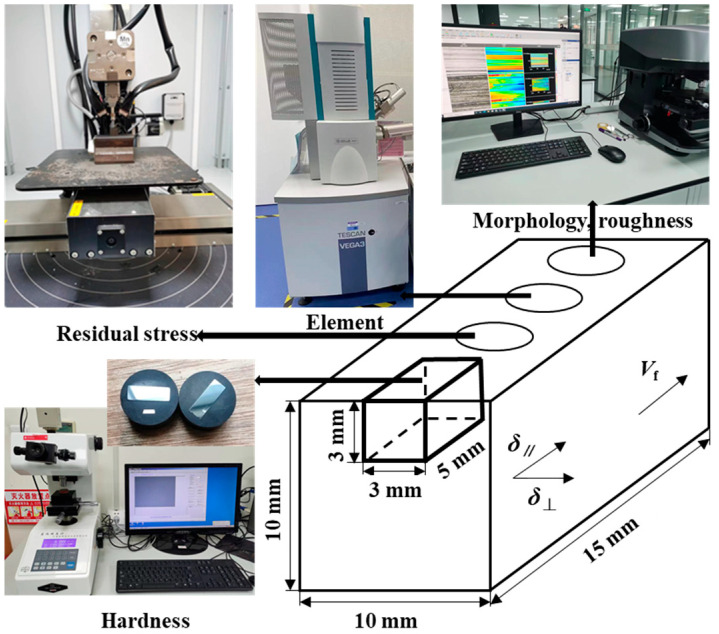
Schematic diagram of surface integrity testing.

The study of the influence of process parameters on the surface integrity of high-strength steels can effectively realize the improvement and control of the surface quality of parts, thus ensuring the service performance of products. As indicated in Figure 2, the VK-X3000-material-type upright laser confocal microscope was used to observe the morphology of the ground surface and measure surface roughness. A scanning electron microscope of the type VEGA 3-XMU (LaB6) was used for the analysis of point elements and subsurface structures of the ground surfaces. A Proto-LXRD-type X-ray stress analyzer was used to detect the residual stress on the surface of the specimen. Cross-sectional metallographic specimens were prepared during the process using a MECAPRESS 3 hot setter and a MECATECH 334 automatic polishing machine.

It is worth noting that, for commonly used objects of 30Cr2Ni4MoV steel, the processing parameters used in the experiment are based on the actual production line of gas turbine rotor manufacturing. In addition, to reduce testing errors, the measurement of grinding process parameters and surface integrity indicators are repeated three times under the same operating conditions and the average value is taken. The calculation and measurement errors are presented in the form of error bars.

## 3. Grindability Analysis

### 3.1. Grinding Force

The magnitude of the grinding force is related to the lifespan of the grinding wheel, the control of the surface roughness of the parts, and the change in the grinding energy. Therefore, the grinding force is an important measurement and reference data for the high-strength steel grinding process.

Material removal is generally evaluated in terms of maximum undeformed chip thickness (*a*_gmax_), as shown in Equation (1). In fact, the smaller the *a*_gmax_, the lower the grinding force. Song et al. [14] believed that this is due to the relatively low material removal rate of individual abrasive particles caused by smaller *a*_gmax_, which reduces mechanical load and grinding force.

(1) $$a_{\mathrm{gmax}}={\left[\frac{4}{CN_{d}}\times \frac{V_{f}}{V_{s}}{\left(\frac{a_{p}}{d_{s}}\right)}^{0.5}\right]}^{0.5}$$

where C is the coefficient related to the half angle of the tip of the effective abrasive grain; *N*_d_ is the density of the effective cutting edge (g/cm^3^); *d*_s_ is the diameter of the grinding wheel (mm).

Additionally, the relationship between the combined grinding force (*F*) and the component forces is given by the following equation [15].

(2) $$F=\sqrt{{\left(F_{x}\right)}^{2}+{\left(F_{y}\right)}^{2}+{\left(F_{z}\right)}^{2}}$$

where *F*_x_, *F*_y_ and *F*_z_ are the axial, radial and tangential grinding forces (N), respectively.

**Figure 3:**
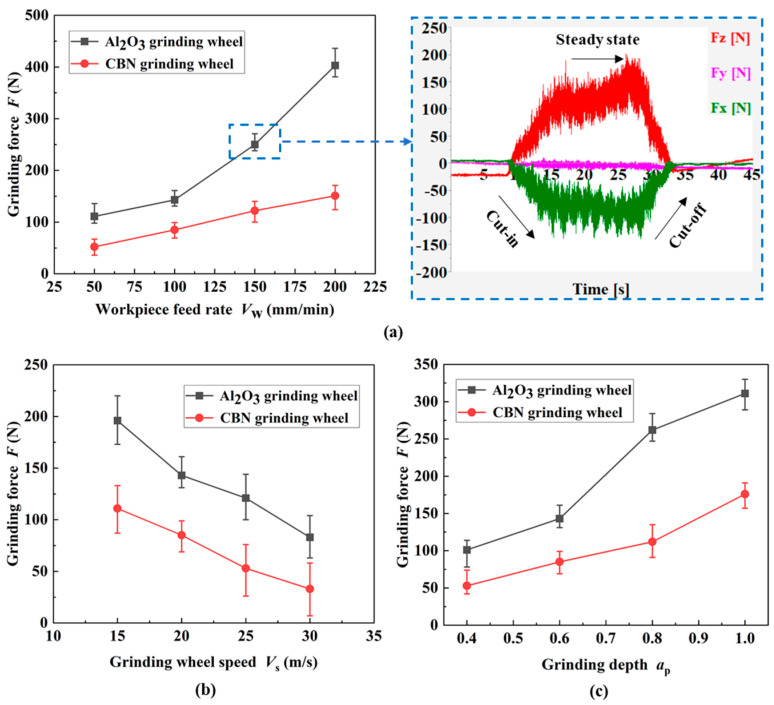
Effect of process parameters on the grinding force ((**a**) *V*_s_ = 20 m/s; *a*_p_ = 0.6 mm; (**b**) *V*_f_ = 100 mm/min; *a*_p_ = 0.6 mm; (**c**) *V*_s_ = 20 m/s; *V*_f_ = 100 mm/min).

As shown in Figure 3, *V*_f_, *V*_s_ and *a*_p_ were set as single variables, each group of tests was repeated three times, and the measurement results were averaged. The effect of process parameters on the *F* of two kinds of grinding wheels was explored and the trend and reasons for the change in *F* were analyzed. The right side of Figure 3a (dotted blue line) shows the acquired signals of each grinding component force, including the cut-in, steady-state and cut-out phases. As indicated in Figure 3a–c, the *F* from both grinding wheels increases almost linearly as the *a*_p_ rises. The reason behind this is that the number of effective abrasive grains on the surface of the grinding wheel involved in material removal ascends, the contact arc length between the abrasive grits and the grinding wheel as well as the *a*_gmax_ increase accordingly, and the *F* increases. As the linear velocity of the grinding wheel gradually increases, both the *F*_x_ and *F*_z_ gradually decrease, and there is a negative correlation between the two. The increase in *V*_f_ leads to an increase in both normal and tangential forces, which are positively correlated. The increase in the linear speed of the grinding wheel reduces the *a*_gmax_, thereby reducing the grinding force [16]. As the workpiece speed increases, both *a*_gmax_ and material removal rate increase, resulting in an increase in *F*_x_ and *F*_z_.

### 3.2. Grinding Temperature

**Figure 4:**
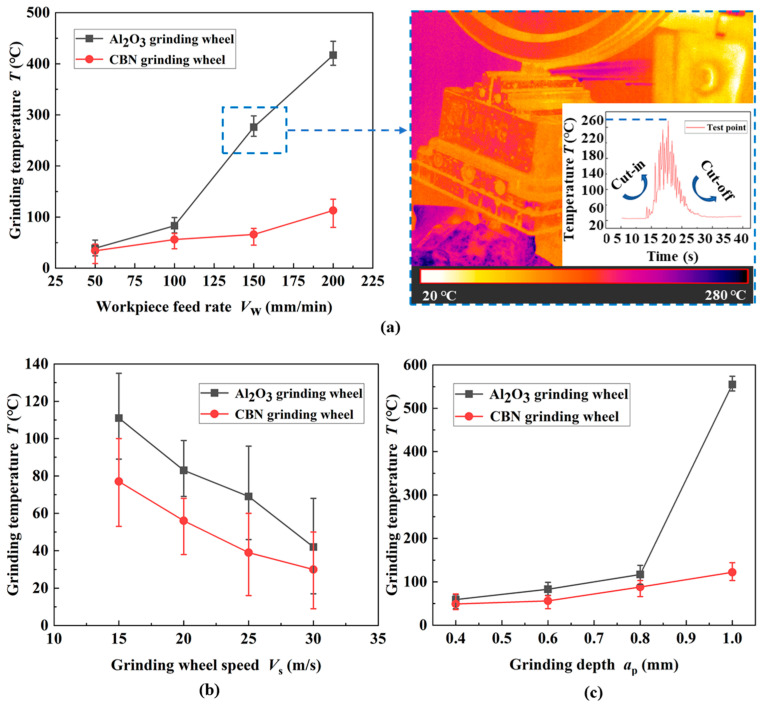
Effect of process parameters on the grinding temperature ((**a**) *V*_s_ = 20 m/s; *a*_p_ = 0.6 mm; (**b**) *V*_f_ = 100 mm/min; *a*_p_ = 0.6 mm; (**c**) *V*_s_ = 20 m/s; *V*_f_ = 100 mm/min).

As illustrated on the right side of Figure 4 (dotted blue line), the highest temperature was found to occur in the area where the grinding wheel cuts out the workpiece. When the grinding wheel first comes into contact with the workpiece, the temperature in the grinding area rises at an extremely fast rate. When entering normal grinding processing, the *T* gradually increases but the heating rate gradually slows down. After processing, the peak temperature rapidly decreases in the short term.

From Figure 4a,c, it can be seen that the *T* rises rapidly with the increase in *V*_w_ and *a*_p_. The reason for this phenomenon is that the elevation of *a*_gmax_ increases the grinding load and generates a large amount of heat. *a*_p_ promotion causes the grinding arc length to increase, and it is difficult for the grinding fluid to enter the friction interface to play a cooling role. The increase in *V*_s_ results in a higher number of effective grits passing through the grinding arc per unit time, and the *a*_gmax_ becomes thinner, which reduces the grinding load. In addition, higher *V*_s_ increases the frequency of heat transfer in the grinding zone. Figure 4b verifies the above statement that the *T* decreases gradually with increasing V_s_. The two types of grinding wheel exhibit the same trend under different machining parameters.

In addition, the *T* of CBN abrasives is lower than that of SG under the same working conditions. This is because the SG abrasive is composed of submicroscopic crystals, which can continuously provide new cutting edges through micro-crushing under pressure and take away the grinding heat with high self-sharpening. CBN abrasive has superior thermal conductivity and abrasion resistance. It can be seen that CBN abrasive grain in the inhibition of high-strength steel grinding thermal damage ability is more prominent and the cold cutting effect is better [3,17].

When the heat flux density in the grit–workpiece contact zone is close to but does not exceed the critical heat flux density, the grinding fluid in the arc region is in the nuclear boiling stage. At this time, the water-based coolant under the action of the workpiece surface temperature is maintained at about 130 ℃ below. As illustrated in Figure 4a,c, when the heat flow density in the grinding arc exceeds the critical value, grinding fluid film boiling occurs, and the accumulated heat causes the surface temperature of the workpiece to rise sharply, thus causing sudden burns.

### 3.3. Grinding Specific Energy

As shown in the following formula, the grinding specific energy refers to the energy used to remove a unit volume of workpiece material, and its change with the grinding time can also be used as a basis for judging the grinding performance [18].

(3) $$E_{s}=\frac{P}{Q_{w}}=\frac{V_{s}\cdot F_{t}}{V_{f}\cdot a_{p}\cdot b}$$

where *E*_s_ is the specific grinding energy; *Q*_w_ is the unit volume of material removed by a single grit; *P* is the work conducted by a single grit to remove a unit volume of material; *b* is the width of the workpiece.

**Figure 5:**
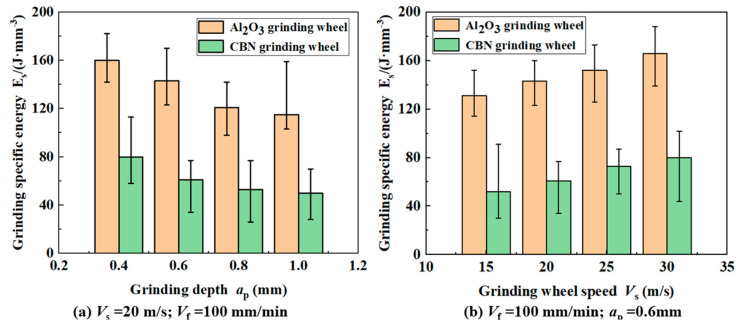
Effect of grinding depth (**a**) and wheel rotating speed (**b**) on grinding specific energy of two kinds of wheel.

As shown in Figure 5a, the *E*_s_ of both grinding wheels for machining high-strength steel decreases with the increase in *a*_p_. The decreasing trend of SG grit gradually becomes slower with the increase in *a*_p_. The grinding *V*_f_ and *V*_s_ remain stable; increasing the *a*_p_ will lead to a reduction in the *a*_gmax_, the energy required to remove the unit volume of material is reduced, and so the *E*_s_ will decrease. After rapid wear of the abrasive grains on the surface of the corundum grinding wheel, the abrasives become blunt and rounded at the tip, increasing the actual negative rake angle. In addition, the shear flow stresses in the metal increase. As a result, there is a tendency for the reduction in the specific energy of grinding to slow down. As shown in Figure 5b, the *a*_p_ and *V*_f_ are kept constant and, after increasing the *V*_s_, the *E*_s_ of the two grinding wheels can show a smooth upward trend. This is due to the increase in *V*_s_; the *a*_gmax_ of the abrasive grain decreases, enhancing the shear strain effect and shear strain rate effect of the material, weakening the thermal softening effect, requiring higher energy to remove the unit volume of material [19]. Calculations and comparisons show that the average grinding ratio of CBN grinding wheels is approximately 33.5% lower than that of SG grinding wheels. The lower the specific energy of grinding means that the grits are more capable of removing material. Although SG grinding wheels are highly self-sharpening, the ultra-high hardness of CBN abrasives makes it easier to maintain a sharp cutting edge.

CBN grinding wheels are characterized by superior wear resistance, high thermal conductivity and unique chemical stability. Compared with corundum abrasives, the grinding performance has been greatly improved and it is more suitable for grinding difficult-to-machine metal materials with high strength and low thermal conductivity.

In summary, during the grinding process of high-strength steel, the increase and decrease trends of *F* and *T* are basically consistent. The source of both *F* and *T* is the elastic-ductile deformation and friction of the workpiece material under the action of abrasive grains, and the grinding force and the grinding heat conducted to the workpiece part affect the surface integrity of the specimen. It can be seen that the basic idea of reducing the thermal damage of high-strength steel grinding can be divided into two aspects. On the one hand, it is to reduce the heat generated when the workpiece interacts with the abrasive grain from the source. On the other hand, it is to start from the conduction link of grinding heat. By strengthening the heat dissipation in the machining arc area, the grinding temperature can be reduced. If economic conditions permit and there is a high demand for part quality, superhard abrasive grinding wheels and corresponding dressing facilities can be equipped [20,21]. Otherwise, cooling methods such as cryogenic minimal-quantity lubrication can be chosen to break through the gas barrier and achieve enhanced heat transfer [22,23]. How to establish effective suppression schemes based on the formation mechanisms of thermal damage in high-strength steel grinding has become an important direction for future scientific development.

## 4. Effects of Thermal Damages on Surface Integrity in Steel Grinding

### 4.1. Surface Morphology and Roughness

**Figure 6:**
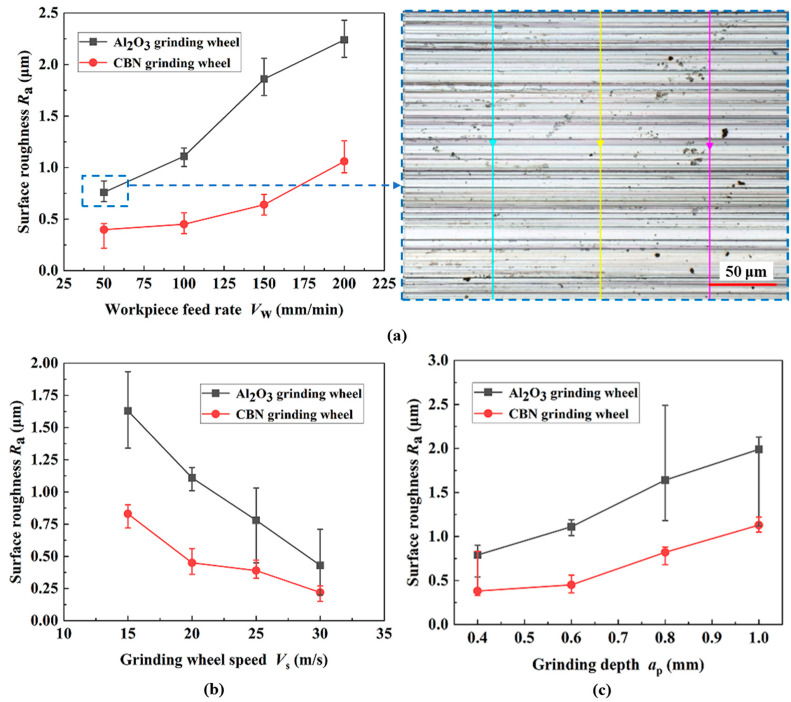
Effects of grinding parameters on surface roughness ((**a**) *V*_s_ = 20 m/s; *a*_p_ = 0.6 mm; (**b**) *V*_f_ = 100 mm/min; *a*_p_ = 0.6 mm; (**c**) *V*_s_ = 20 m/s; *V*_f_ = 100 mm/min).

Grinding surface roughness is one of the most important parameters for surface integrity, and the arithmetic mean deviation *R*_a_ of the profile is commonly used for evaluation and characterization. Similarly, the effect of machining parameters on *R*_a_ was studied by controlling a single variable. As shown on the right side of Figure 6a (dotted blue line), a stable machining area was selected and the *R*_a_ of the ground surface was measured and averaged at three locations along the *V*_f_ direction (Multi-colored arrows represent measurement paths).

As shown in Figure 6a, the *a*_gmax_ and the ductile deformation of a single grit increase with the ascent of the *V*_f_. Furthermore, the cutting effect of abrasive particles becomes stronger while the plowing and scratching effects become weaker. As a result, the *R*_a_ rises in the vertical grinding direction. As the linear speed of the grinding wheel increases, the *a*_gmax_ corresponding to the cutting action of a single abrasive grain decreases, the degree of ductile deformation of the chips decreases, and the *F* decreases. Meanwhile, the number of abrasive grains acting on the end of the grinding arc per unit of time increases, and the plowing and scratching effect of the abrasive grains here is weakened [24]. As a result, the surface texture of the specimen becomes more uniform and the corresponding *R*_a_ value decreases, as displayed in Figure 6b. Due to the increase in grinding depth, the undeformed chip thickness and length of a single abrasive grain will increase, exacerbating the ductile deformation of the chips. The workpiece material bulges on both sides, forming irregular plow grooves, resulting in a decrease in surface quality and an increase in corresponding grinding roughness, as revealed in Figure 6c.

To visualize the influence of thermal damage defects on the surface properties of the specimen during creep-feed grinding of high-strength steel, the morphology and texture of the ground surface were measured and analyzed, respectively.

**Figure 7:**
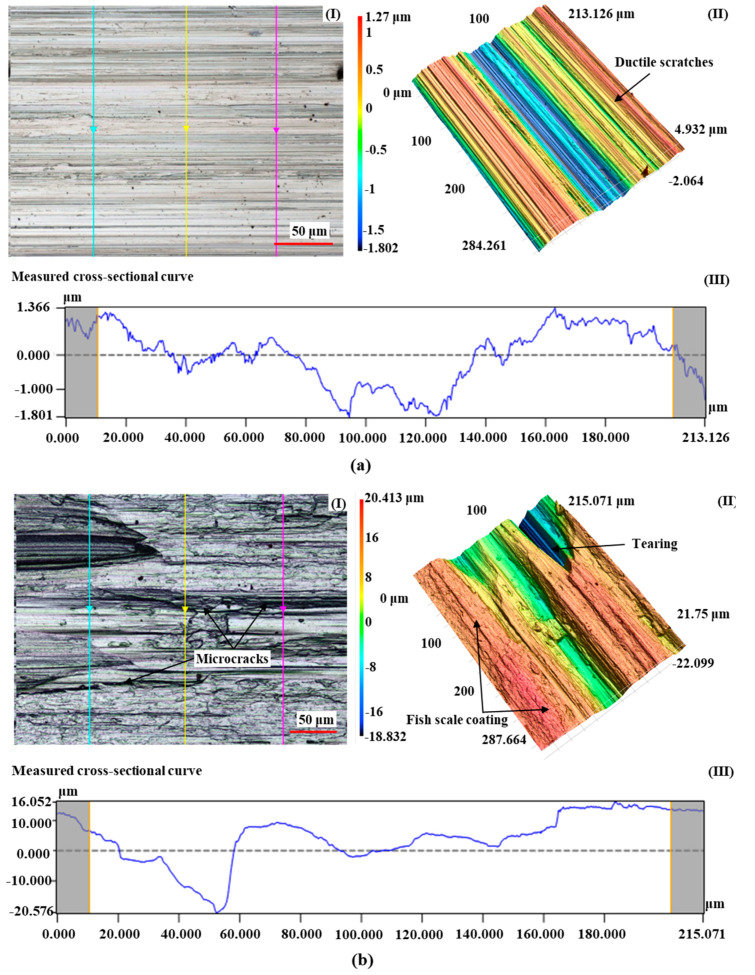
Effect of grinding thermal damages on surface morphology and *R*_a_ of high-strength steel. ((**a**) Burn-free workpiece machined by CBN grinding wheel; (**b**) Burn-out workpiece machined by Al_2_O_3_ grinding wheel).

As shown in Figure 7, the surface morphology (I), three-dimensional profile (II) and cross-section measurement curve (III) along the feed direction of the high-strength steel processed by the two grinding wheels were comparatively analyzed by using the same combination of machining parameters (*V*_s_ = 20 m/s; *V*_f_ = 100 mm/min; *a*_p_ = 1 mm). It can be seen that, compared with the Figure 7a(I), the Figure 7b(I) exhibits alternating oxidation discoloration of bright spots and gray on the surface. As a rule, the darker the color, the more severe the burns. In addition, due to the combined effect of strong plastic deformation and high grinding temperature, the pattern of corundum abrasive wheel grinding is irregular, the two sides of the plastic bulge are not uniform and there are a lot of abrasive chips and adherents on the processed surface.

Due to the high strength and poor thermal conductivity of the material, the force thermal load in the contact area between SG abrasives and workpiece material is large, and the surface of the workpiece is prone to a fish-scale-like coating phenomenon. The rapid cooling of the surface of high-strength steels after the application of water-based grinding fluids produces an increase in the amount of secondary quenched martensite, a change in the lattice and a reduction in volume. However, the subsurface layer becomes a lower hardness tempered organization due to slow cooling, which increases the tendency of residual tensile stresses and microcracks on the surface of the workpiece, as displayed in Figure 7b.

In summary, under the same process parameter combination (*V*_w_ = 50 mm/min; *V*_s_ = 20 m/s; *a*_p_ = 0.6 mm), the specimens processed with the SG grinding wheel exhibit thermal damage defects, while the CBN grinding wheel produces burn-free workpieces. Therefore, based on these two sets of experimental results, the effects of grinding thermal damage on the hardness and residual stress of high-strength steel are analyzed in Section 4.2 and Section 4.3, respectively.

### 4.2. Surface Residual Stresses

**Figure 8:**
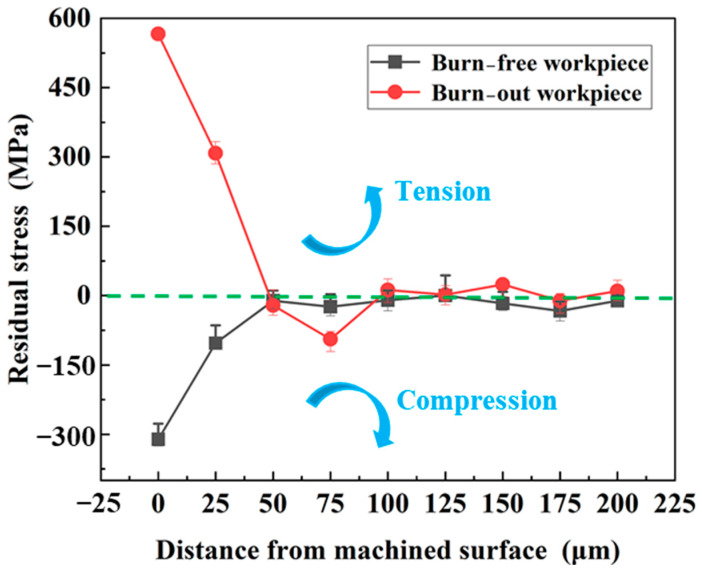
Effect of grinding thermal damages on the residual stress of the skin layer of high-strength steel (*V*_s_ =20 m/s; *V*_f_ =100 mm/min; *a*_p_ =1 mm).

As shown in Figure 8, the residual stresses at different depths of the abraded workpiece were measured at 25 μm intervals by electrolytic corrosion. It can be found that the surface of the burn-out workpiece shows a large tensile stress, nearly 300 MPa, at a depth of 50 μm. Nevertheless, the stress state on the surface of a burn-free specimen is always compressive stress, which gradually reaches a constant at a depth of 50 μm.

It is generally believed that, under appropriate cooling conditions, the mechanical ductile deformation of the material caused by chip removal and the thermal ductile deformation caused by grinding heat are the main reasons for the formation of residual stresses in grinding. The residual stress triggered by mechanical ductile deformation is generally compressive stress. The residual stress caused by thermal ductility deformation manifests as tensile stress [24]. When grinding heat can produce thermoplastic deformation on the surface of the specimen, high-strength steel will experience thermal expansion and an increase in material volume. However, the ductile deformation of materials is irreversible and, under the action of elastic deformation, the lower-layer material will suppress the shrinkage of the upper-layer material, thereby forming residual tensile stress.

### 4.3. Surface Microhardness

**Figure 9:**
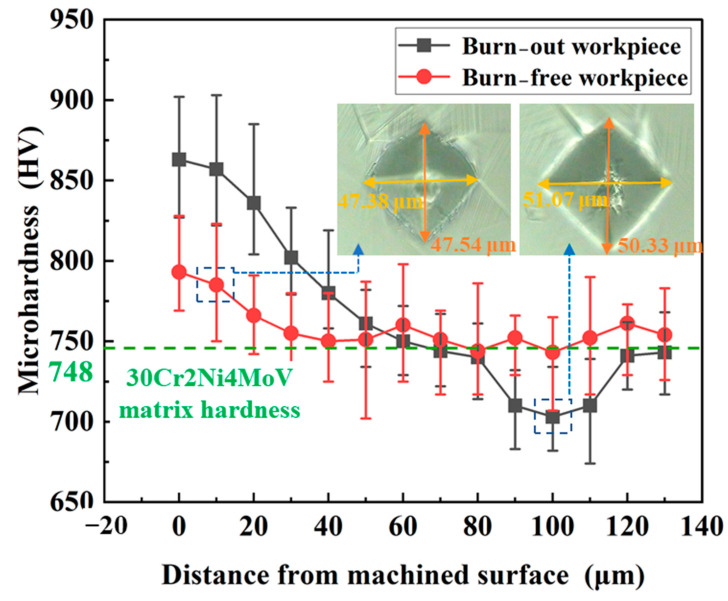
Effect of grinding thermal damages on the microhardness of the skin layer of high-strength steel (*V*_s_ = 20 m/s; *V*_f_ = 100 mm/min; *a*_p_ = 1 mm).

As shown in Figure 9, the thickness of the material layer for work hardening and heat softening ranges from a few micrometers to several tens of micrometers. The microhardness of high-strength steel gradually recovers to the hardness of the workpiece matrix as the depth of the subsurface layer increases. The measurement results of indentation size are shown in the figure, and the matrix hardness of 30Cr2Ni4MoV steel is marked as a control.

CBN grinding wheels possess the desired retention of grit sharpness and the material removal rate is well controlled. The grinding temperature at this time is lower than the recrystallization temperature of high-strength steel. The slip and distortion of the crystal lattice makes the surface material strength and hardness increase. The SG grit wheel is severely passivated, and the operating temperature under conditions of excessive material removal rate exceeds the phase transition temperature or recrystallization temperature of the specimen. The organization near the region will gradually undergo transformation, the reinforcing phase is decomposed and the hardness decreases rapidly, resulting in softening of the surface layer. The processing temperature of the subsurface area is lower than the softening temperature of the material. In addition, due to plastic deformation and strain failure, the material there will exhibit a cold deformation strengthening effect and microhardness increases.

In short, the abrasive grits interact with the workpiece in the grinding arc, and the large ductile deformation causes the grains in the surface layer of the steel to slip and twin and its hardness will increase significantly. Due to friction and the ductile deformation of the chips, high processing temperatures and even burns are generated and, if the cooling is insufficient, the hardness of high-strength steel decreases as a result of phase transformation. The above factors are coupled with each other to determine the hardness of the workpiece.

### 4.4. Surface Energy Spectrum

As detailed in Table 1, the addition of alloying elements such as Cr, Ni, Mo, V, Mn, etc., to steel reduces the thermal conductivity of the components and deteriorates their grindability, making them a typical difficult-to-machine material. It can be seen that elemental properties play an important role in the mechanical properties of components. It is of profound significance to clarify the effect of grinding thermal damage on the type and content of elements in high-strength steels.

**Figure 10:**
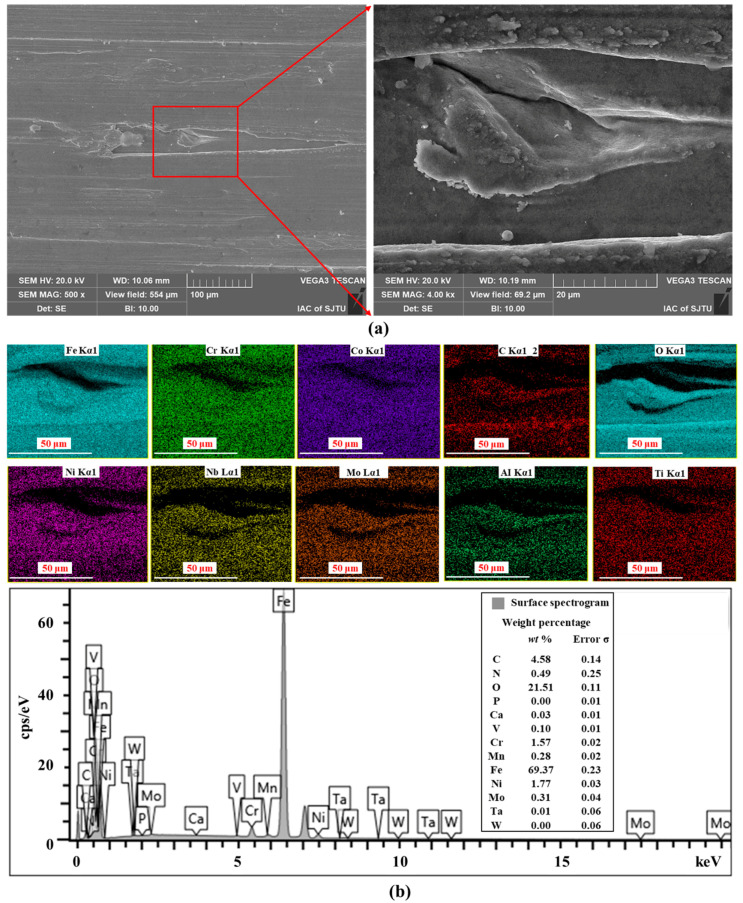
Effect of grinding thermal damages on the type and content of elements in the micro-zone of high-strength steel ((**a**) Secondary electron morphology of the ground surface; (**b**) Surface spectrogram of the ground surface).

With the help of different X-ray quantum energies, the surface of the burn-out workpiece surface (*V*_s_ = 20 m/s; *V*_f_ = 100 mm/min; *a*_p_ = 1 mm) of high-strength steel caused by SG abrasive machining was face scanned, and the images of the distribution of the content of each element were obtained, as shown in Figure 10. The grinder adhesive wear is severe in the creep-feed grinding mode, and the micro-fragmentation property of corundum abrasive allows more dislodged crystal debris to adhere to the machined surface. The superposition of the elemental species of the final material micro-zone composition occurs. By comparison, it can be found that oxygen elements appear on the burn-out surface and their content is quite high. This indicates that there are more oxides at this time and the metallographic structure of the sample changes at high temperature. This is also the essential reason for the thermal damage caused by changes in the properties of high-strength steel parts.

### 4.5. Surface Organizational Structure

**Figure 11:**
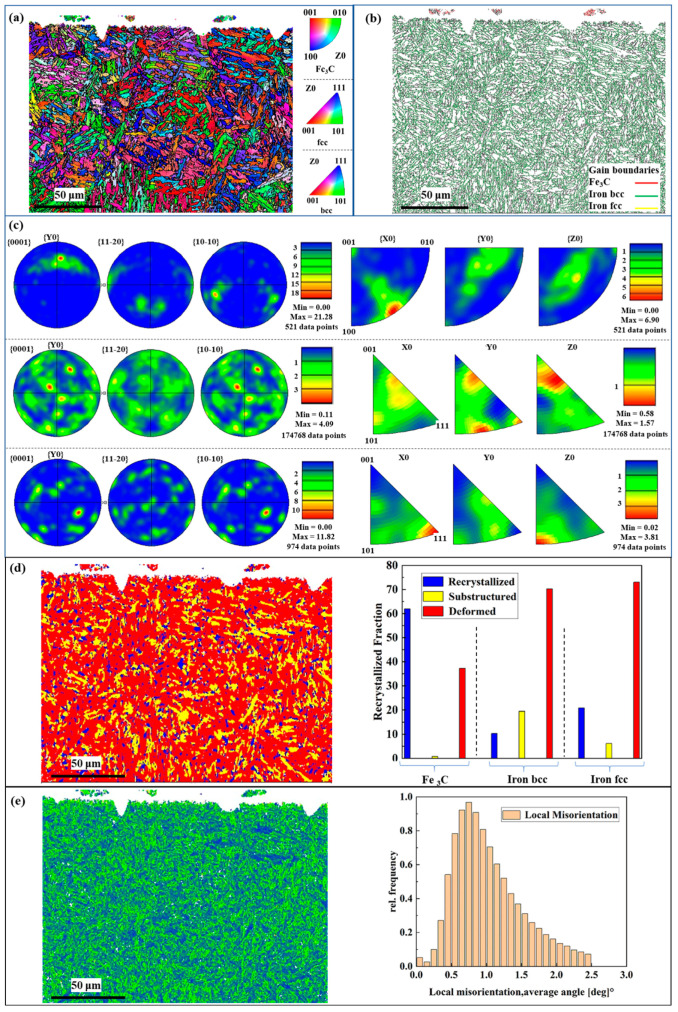
Grain size and shape of high-strength steel burn-out workpieces and their polar and antipolar plots (**a**–**c**); phase reversion, recrystallization, deformation and its percentage (**d**); local misorientation diagram and KAM statistical histograms (**e**).

The high grinding temperature will not only change the surface properties of the specimen but also change the organization of its subsurface layer. Samples processed by SG abrasives at the parameter combination of (*V*_s_ = 20 m/s; *V*_f_ = 100 mm/min; *a*_p_ = 1 mm) were analyzed for phase identification, dimensions, morphology and orientation. As shown in Figure 11a–c, when the instantaneous temperature of the surface layer during grinding reaches the phase transformation temperature of 30Cr2Ni4MoV, the surface material of the workpiece will be reaustenitized. According to the principle of high-temperature deformation heat treatment, the austenite grain will be refined, the dislocation density within the grain is higher and there is the occurrence of dynamic recrystallizing of the nucleation of more parts [25]. Therefore, high-temperature austenite deformation, fragmentation will form a dense structure. Under the action of the coolant, grinding-surface high-speed cooling results in the surface layer of the material being too late for austenite recrystallization, resulting in small secondary quenching martensite organization; in addition, the austenite-to-martensite transformation is not completely completed and the white layer is still retained in the residual austenite organization. Therefore, high-strength steel in the corundum abrasive thermal damage is caused by the action of the secondary quenched martensite, granular carbides and residual austenite composition. The increased material deformation caused by high temperature and its thermal softening satisfies the necessary conditions for recrystallization of high-strength steels. While the recrystallized grain boundaries are the origin of crack sprouting, the crack extension direction will be approximately the same with the recrystallized grain boundaries, bending and extending to the machined surface, as shown in Figure 11d. As shown in Figure 11e, the heat-damaged workpiece shows grain refinement and the peak of regional orientation difference is shifted to the right, indicating increased plastic deformation.

From the above analysis, it can be concluded that the formation of surface integrity in high-strength steel grinding is an extremely complex process formed by the interaction of multiple factors. Surface stresses during grinding of high-strength steel include thermal, mechanical and organizational stresses. When the sum of the maximum tensile stresses exceeds the yield strength limit of the specimen, the ground surface is torn and grinding cracks appear. The surface temperature of the specimen drops rapidly under the action of the coolant after grinding but the temperature of the internal material remains high. This allows the internal material to continue to expand and generate tensile stresses. At the same time, the surface organization changes from austenite to secondary quenched martensite, while the subsurface layer can only form a high-temperature tempered organization due to the large temperature gradient and short time of action. These physical changes drive the alteration of the surface integrity of high-strength steels. However, the changes in these factors are largely due to the variability of process conditions. Clarifying the influence of process conditions on surface integrity is the theoretical basis for improving the surface integrity of high-strength steel during grinding.

## 5. Conclusions

In this paper, a creep-feed grinding test of high-strength steel is carried out to study the mechanisms of grinding wheel performances and process parameters on the grindability of high-strength steel and to analyze the effects of thermal damage defects on the surface integrity of high-strength steel.

- High-strength steel grinding generates a large amount of heat during the material removal process due to its high strength and toughness, coupled with difficulties in heat dissipation. The heat accumulation in the machining arc zone results in a heat flux density exceeding the critical value, and the grinding fluid loses its heat transfer ability. Instantaneous high temperature causes changes in the metallographic structure of the workpiece surface, resulting in thermal damage defects.
- Within the parameters of this experiment, the CBN grinding wheel reduces the grinding specific energy by about 33% compared to the SG grinding wheel and is able to control the surface roughness below 0.8 µm. The weight percentage of oxygen element in the burn-out workpiece is 21% and the thickness of the metamorphic layer is about 40 µm.
- The quantitative relationship and influence mechanisms of machine parameters on grinding force, grinding temperature and specific energy of grinding are revealed. The process parameters determine the procedure parameters and surface quality of high-strength steel grinding by adjusting the maximum undeformed chip thickness.
- The ultra-high wear resistance and thermal conductivity of CBN abrasive particles are superior to the self-sharpening of SG abrasive in improving the grindability and surface integrity of high-strength steel.
- The effects of thermal damage on the surface integrity of high-strength steel grinding are mainly reflected in the appearance of oxidative discoloration, fish-scale coating texture, microcracks, crushed craters, residual tensile stresses, reduced hardness, transformation of the metallurgical organization and greater surface roughness.

## Figures and Tables

**Table 1:** Original composition of 30Cr2Ni4MoV high-strength steel.

Element	C	Ni	Mn	Si	P	Mo	Cr	V	S
Mass (%)	0.24–0.34	1.8–2.2	0.5–0.8	0.17–0.37	≤0.035	0.3–0.5	1.35–1.65	0.07–0.12	≤0.035

**Table 2:** Thermo-mechanical property parameters of high-strength steel and abrasives.

	30Cr2Ni4MoV	Al_2_O_3_ Grits	CBN Grits
Density *ρ* (kg/m^3^)	7850	3980	3480
Elastic modulus *E* (Gpa)	190	530	900
Poisson ratio *v*	0.3	0.2	0.128
Thermal conductivity *λ* (W/m·K)	42.5	29.31	240
Specific heat capacity *c* (J/Kg·K)	490	780	506
Thermal expansion coefficient *a* (1/°C)	12.2 × 10^−6^	7.7 × 10^−6^	3.5 × 10^−6^
Melting temperature *T*_m_ (°C)	1420	2054	3000
Yield strength *σ*_s_ (N/mm^2^)	785	300	/
Tensile strength *σ* (N/mm^2^)	930	160	/
Vickers hardness HV (N/mm^2^)	748	2100	8820

**Table 3:** Feature parameters of experimental grinding stones.

Index	Parameter	
Grinding tool type	Corundum grinding wheel	Superabrasive grinding wheel
Abrasive composition	*α*-Al_2_O_3_	CBN
Binding agent type	Ceramics	Nickel
Abrasive holding method	Sinter	Electroplate
Grit size	80#	80#
Grain density	100%	100%
Inner diameter (mm)	127	127
Outer diameter (mm)	400	400
Axial thickness (mm)	50	50

**Table 4:** Parameter settings for high-strength steel grinding tests.

Machining Parameters (Units)	Values
Processed material	High-strength steel (30Cr2Ni4MoV)
Workpiece feed rate, *V*_f_ (mm/min)	50, 100, 150, 200,
Wheel Speed, *V*_s_ (m/s)	15, 20, 25, 30
Grinding depth, *a*_p_ (mm)	0.4, 0.6, 0.8, 1.0
Feed distance, *l_f_* (mm)	20
Grinding method	Surface grinding; Down grinding
Coolant conditions	Baso Vasco 7000 water-soluble cutting fluid; Pressure 8 × 10^8^ pa, flow rate 120 L/min.
Grinding wheel dressing conditions	Diamond disk; dressing speed 200 mm/min; dressing depth 0.2 mm

## Data Availability

Data are contained within the article.
